# Professional Advertisements

**Published:** 1878-10

**Authors:** 


*To the Editor of the Chicago Medical Journal and Examiner:*

One of your correspondents, "E. Ingals, M. D.," in an article in your September number, in which he arraigns a goodly number of the medical profession of this city for alleged violation of the medical code, says:

"An advertisement appears day after day in the most widely circulated papers of Chicago, signed by more than a dozen well-known physicians of the city, who have united in the Herculean task of informing a distressed and waiting public of whom it may purchase a pair of spectacles."

Can this be so? Did the Doctor read the advertisement correctly? Did he put on *his* spectacles? Or, is the defect at the *other end* of his optic nerve? What are the real facts about this famous advertisement? Simply these:—A clever artisan publishes in the daily papers his business card, in which he states that he is recommended by certain gentlemen, giving their names, and appending, on his own responsibility, their titles and public position. *This is the whole case!*

Now, as my name is in the list, I desire to say that I hold myself strictly amenable to the code of ethics adopted by the American Medical Association, and I defy your correspondent to cite a single section of that code, the letter or spirit of which I have violated. But I suspect that the doctor has read the code about as intelligently as he read the advertisement which has disturbed him so much, and against which he has done such Quixotic warfare. MOSES GUNN.

---

CHICAGO, Sept. 6th, 1878.

*Editor of the Chicago Medical Journal and Examiner:*

I must confess to a feeling of disappointment as I concluded my perusal of the correspondence in the last number of the CHICAGO MEDICAL JOURNAL AND EXAMINER. Glancing down the pages occupied by your distinguished correspondent, I was anxiously looking for something that should make me feel "the crimson in my cheek;" and, at last, I thought it was really coming, as the wonderful advertisement of the surgeon-dentist began to unfold its "lingering length" before my view. Now for a spicy narrative of a convulsion in the British Medical Association, I thought. I expected to read of nothing less than the death of the venerable Dr. Heberden, occasioned by mortification at sight of the name of John Hunter in the advertisement of a quack. Undoubtedly, we should be told of an outbreak of acne rosacea upon the proboscis of Sir Charles Bell, excited by a violent determination of "crimson" to the part. Beyond all question we should be informed of the manner in which Sir Astley Cooper and Mr. Abernethy took measures to punish this "offense to the (*sic*) honor and dignity of the medical profession" by getting up in the College of Surgeons, "demanding an explanation," and moving a reference of the case to the Royal Society of London, and to the Academy of Sciences in Paris.

But, "oh, most lame and impotent conclusion!" there was nothing of that sort; and I was compelled to lay down my journal with the conviction that if Dr. Heberden and Dr. Bell, and Messrs. Cooper and Abernethy ever cared to notice the document in question, they could only have smiled a little at the ingenuity of an enterprising dentist, and then calmly resumed those studies which have made their names immortal.

Allow me to suggest that if some of your correspondents would keep their minds as seriously occupied as these our predecessors did, they would find no leisure for the work of magnifying mole-hills into mountains, nor feel tempted to violate the code of ethics by thoughtless—let us hope unintentional—misrepresentation of their best friends. As for the allegations of your correspondent, it really is not worth while to take the time of your readers by a formal exposure of their utterly subjective origin and character. HENRY M. LYMAN.

